# Treatment with a Small Molecule Mutant IDH1 Inhibitor Suppresses Tumorigenic Activity and Decreases Production of the Oncometabolite 2-Hydroxyglutarate in Human Chondrosarcoma Cells

**DOI:** 10.1371/journal.pone.0133813

**Published:** 2015-09-14

**Authors:** Luyuan Li, Ana C. Paz, Breelyn A. Wilky, Britt Johnson, Karina Galoian, Andrew Rosenberg, Guozhi Hu, Gabriel Tinoco, Olaf Bodamer, Jonathan C. Trent

**Affiliations:** 1 Division of Hematology and Oncology/Department of Medicine, University of Miami Miller School of Medicine, Miami, Florida, United States of America; 2 Sylvester Comprehensive Cancer Center, University of Miami Miller School of Medicine, Miami, Florida, United States of America; 3 Sheila and David Fuente Graduate Program in Cancer Biology, University of Miami Miller School of Medicine, Miami, Florida, United States of America; 4 Department of Human Genetics, University of Miami Miller School of Medicine, Miami, Florida, United States of America; 5 Department of Orthopaedic Surgery, University of Miami Miller School of Medicine, Miami, Florida, United States of America; 6 Department of Pathology, University of Miami Miller School of Medicine, Miami, Florida, United States of America; Queen's University Belfast, UNITED KINGDOM

## Abstract

Chondrosarcomas are malignant bone tumors that produce cartilaginous matrix. Mutations in isocitrate dehydrogenase enzymes (IDH1/2) were recently described in several cancers including chondrosarcomas. The IDH1 inhibitor AGI-5198 abrogates the ability of mutant IDH1 to produce the oncometabolite D-2 hydroxyglutarate (D-2HG) in gliomas. We sought to determine if treatment with AGI-5198 would similarly inhibit tumorigenic activity and D-2HG production in IDH1-mutant human chondrosarcoma cells. Two human chondrosarcoma cell lines, JJ012 and HT1080 with endogenous IDH1 mutations and a human chondrocyte cell line C28 with wild type IDH1 were employed in our study. Mutation analysis of IDH was performed by PCR-based DNA sequencing, and D-2HG was detected using tandem mass spectrometry. We confirmed that JJ012 and HT1080 harbor IDH1 R132G and R132C mutation, respectively, while C28 has no mutation. D-2HG was detectable in cell pellets and media of JJ012 and HT1080 cells, as well as plasma and urine from an IDH-mutant chondrosarcoma patient, which decreased after tumor resection. AGI-5198 treatment decreased D-2HG levels in JJ012 and HT1080 cells in a dose-dependent manner, and dramatically inhibited colony formation and migration, interrupted cell cycling, and induced apoptosis. In conclusion, our study demonstrates anti-tumor activity of a mutant IDH1 inhibitor in human chondrosarcoma cell lines, and suggests that D-2HG is a potential biomarker for IDH mutations in chondrosarcoma cells. Thus, clinical trials of mutant IDH inhibitors are warranted for patients with IDH-mutant chondrosarcomas.

## Introduction

Chondrosarcomas are the second most common primary malignancy of bone and are defined by the production of hyaline cartilaginous matrix. Approximately 90% of chondrosarcomas are the conventional subtype and are composed of hyaline and/or myxoid cartilage. The remaining 10% includes dedifferentiated, mesenchymal, and clear cell subtypes that have distinctive clinicopathologic features [1]. Conventional chondrosarcoma is classified as central, peripheral, and periosteal subtypes according to anatomic location, and by grade, with 90% of conventional chondrosarcomas being low or intermediate grade [2]. Currently, surgery is the mainstay of therapy for most patients with localized chondrosarcoma. Chemotherapy is generally ineffective in conventional chondrosarcoma though it is utilized for mesenchymal and dedifferentiated subtypes. Thus, new systemic therapies are urgently needed for unresectable, metastatic or refractory disease.

**Fig 1:**
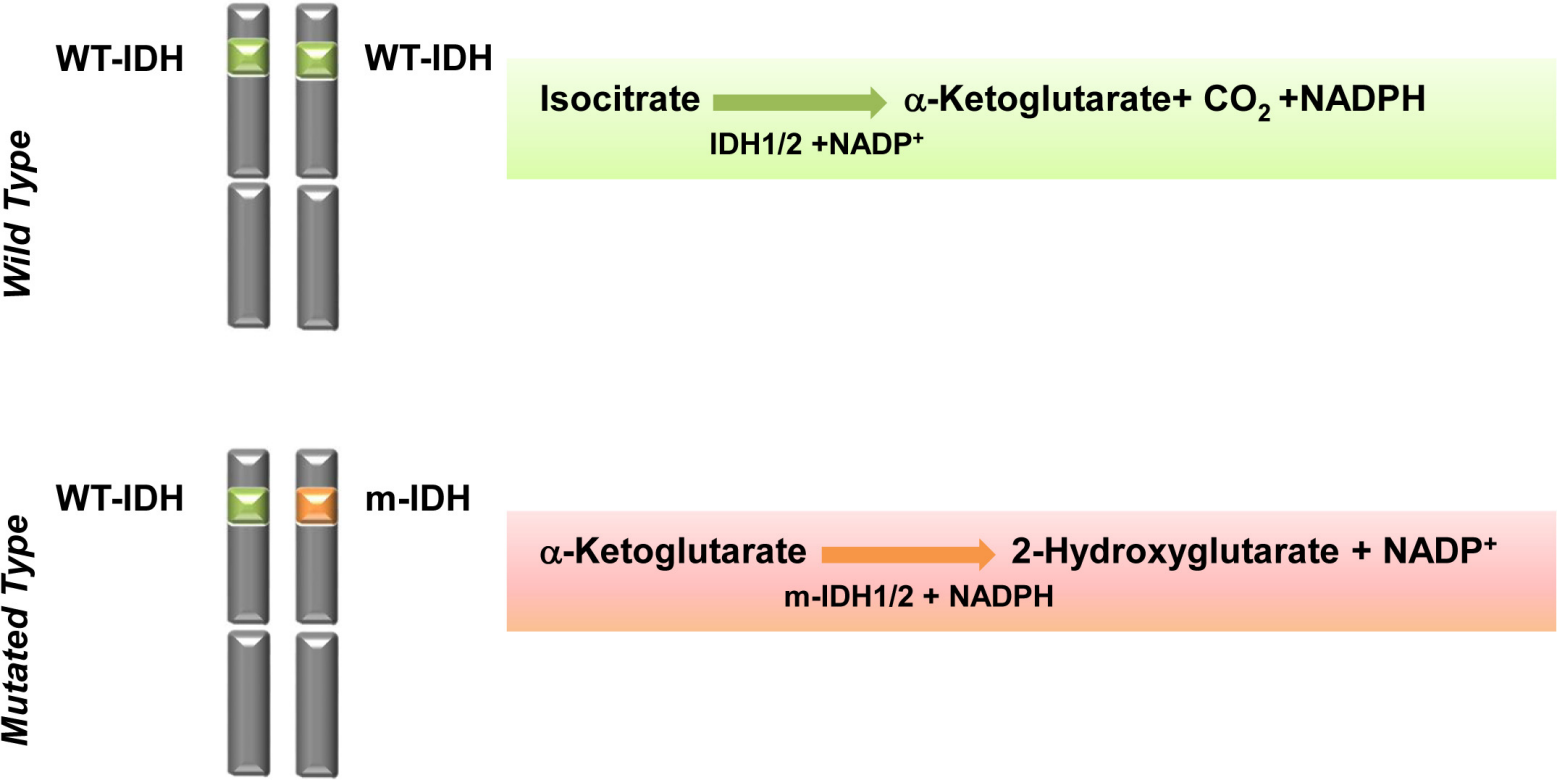
Schema of Altered Metabolic Pathways in IDH 1/2 Mutations. Wild type IDH1/2 uses NADP^+^ as a cofactor and catalyzes the oxidative decarboxylation of isocitrate, producing α-ketoglutarate (α-KG), NADPH and CO_2_. Mutated *IDH* harbors a heterozygous mutation. These mutations occur in a single allele, leading to the inability of enzyme to convert isocitrate into -KG and instead, reduction of α-KG into an oncometabolite, the (D)-enantiomer of 2-hydroxyglutarate (D-2HG).

Isocitrate dehydrogenase (IDH) is an enzyme that catalyzes the oxidative decarboxylation of isocitrate, producing α-ketoglutarate (α-KG), NADPH / NADH and CO_2_. Humans have 3 distinct IDH subtypes. IDH1 and IDH2 are homodimeric enzymes that employ NADP^+^ as a cofactor and localize to the cytoplasm and peroxisomes (IDH1) and mitochondria (IDH2), respectively [3]. IDH3 is a heterotetrameric enzyme which localizes to the mitochondria and utilizes NAD^+^ as a cofactor. Mutations in IDH were recently described in several tumor types including glioma [4–6], acute myeloid leukemia (AML) [7–9] and as well as thyroid [10], breast adenocarcinoma [11] colorectal and prostate carcinomas, and B cell lymphoma [12]. Notably, IDH mutations have also been found in numerous cartilaginous neoplasms, including 71% of conventional chondrosarcomas and 57% of dedifferentiated chondrosarcomas, as well as enchondromas, sporadic central cartilaginous tumors, and periosteal chondromas [1, 3, 13, 14]. Mutations result in a single arginine (R) residue substitution in IDH1 R132 and in IDH2 R172, as well as an occasional mutation of IDH2 R140 in myeloid malignancies [15–19]. These mutations occur in a single allele, leading to the inability of enzyme to convert isocitrate into -KG and instead, reduction of α-KG into an oncometabolite, the (D)-enantiomer of 2-hydroxyglutarate (D-2HG) [16] (Fig 1). 2HG is normally present at low levels in cells, readily interconverted by 2HG dehydrogenase to -KG [20–24]. It was reported that patients with the inherited metabolic disorder 2-hydroxyglutaric aciduria disease caused by 2HG dehydrogenase deficiency accumulate 2HG and have an elevated risk of developing malignant brain tumors [25]. Similarly, dramatically elevated levels of D-2HG have been found in IDH-mutated gliomas [16], cartilage tumors, AML [17] and breast adenocarcinoma [11, 18]. All of the evidence indicates excess D-2HG accumulation produced by mutated IDH contributes to the formation and malignant progression of tumors though the mechanism remains unclear.

Currently, it is believed that 2HG, structurally similar to α-KG, competitively inhibits α-KG-dependent dioxygenases such as TET methyl cytosine hydroxylases and histone lysine demethylases (KDM) that regulate the methylation state of DNA and histone, respectively, and control gene expression epigenetically [3, 26–32]. The induced hypermethylated phenotype has been reported in leukemias, gliomas and cartilaginous tumors [14, 26, 27, 32–34]. It is also thought that 2HG may stabilize HIF1α by competitively inhibiting α-KG-dependent prolyl hydroxylases that facilitate the proteasomal degradation of HIF1α. This activates HIF1α signaling pathways, promoting angiogenesis and tumorigenesis [3, 31, 35–37].

Recently, a new compound, AGI-5198, was identified in a high-throughput screen against mutated R132H-IDH1 enzyme by Agios Pharmaceuticals. In gliomas, this mutant IDH1 inhibitor prevents the mutant enzyme from producing D-2HG in a dose-dependent manner, delays tumor growth, and promotes differentiation [38]. There is no published information regarding the effects of this compound on IDH1-mutant chondrosarcoma cells. Our aim is to determine whether mutant IDH1 inhibitor AGI-5198 exposure alters the tumor phenotype or D-2HG production in IDH1-mutant human chondrosarcoma cell lines. Additionally, as further proof of principle, we sought to determine whether plasma and urine D-2HG levels in an IDH-mutant chondrosarcoma patient differed pre- and post-resection of the tumor. Our results showing the decrease in D-2HG and anti-tumor activity following treatment with AGI-5198 in IDH-mutant chondrosarcoma cells support inclusion of chondrosarcoma patients into ongoing clinical trials of mutant IDH inhibitors for solid tumors.

## Material and Methods

### Cell culture

We utilized two human chondrosarcoma cell lines previously reported to harbor IDH1 mutations: JJ012 [39], a central chondrosarcoma cell line (IDH1 R132G) and HT1080 [40], a dedifferentiated chondrosarcoma cell line (IDH1 R132C). As a normal control, we used C28 [41], a human chondrocyte cell line with wild type IDH1. JJ012 and C28 were kindly provided by Dr. Karina Galoian and HT1080 was obtained from ATCC (ATCC^R^ CCL-121, USA). HT1080 and JJ012 cell lines were grown in RPMI-1640 medium (Lonza, USA) supplemented with 10% fetal bovine serum (FBS) (Gemini, USA) and 1% Penicillin/Streptomycin (Corning Cellgro, USA). C28 cell line was grown in 1:1 DMEM/F12 medium (HyClone, USA) supplemented with 10% FBS and 1% Penicillin/Streptomycin. All cell lines were maintained at 37°C in a 5% CO_2_ incubator.

### Mutation analysis

#### Cell lines

Mutation analysis for *IDH1* on early passage of the three cell lines was performed by means of polymerase chain reaction (PCR) amplification and direct sequencing of DNA. This was done at the Oncogenomics Core Facility, University of Miami Miller School of Medicine. Briefly, DNA was isolated from 4x10^6^ cells using DNeasy Blood and Tissue Kit (Qiagen, Germany) according to the manufacturer’s instructions. A fragment of 122 base pair length including the *IDH1* codon 132 was amplified using the sense primer IDH1F CGGTCTTCAGAGAAGCCATT and the antisense primer IDH1R CACATTATTGCCAACATGAC. Each PCR reaction contained 100 ng of DNA, 10 pmol of sense and antisense primers, 50 mM of MgCl_2_, 10 mM dNTP, 10X NH_4_ and 0.25 μl of Taq DNA Polymerase in a total volume of 25 μL (Bioline, USA). A denaturing step at 94°C for 3 min and 31 cycles with denaturing at 94°C for 30 s, annealing at 60°C for 30 s and extension at 72°C for 20 s were used. PCR products were purified using QIAquick PCR purification Kit (Qiagen, Germany) according to the manufacturer’s instructions. Purified products were sequenced by Sanger sequencing using the same primer as in PCR reaction, and the resulting sequences were analyzed using Finch TV software program (Seattle, WA, USA).

#### Patient samples

Blood, tumor, and urine samples were obtained from a patient with dedifferentiated chondrosarcoma after obtaining informed written consent as per an ongoing IRB-approved sarcoma tissue banking protocol at Sylvester Comprehensive Cancer Center (Study #20121060, Principal Investigator, Jonathan C. Trent). IDH-mutation testing was previously performed for clinical purposes on the patient’s tumor by the Knight Diagnostic Laboratories at Oregon Health and Science University (Portland, OR). Blood and urine samples were obtained prior to and one week following surgical resection of the patient’s chondrosarcoma. Plasma was isolated by centrifugation and all samples were stored at -80°C until analysis.

### Measurement of 2HG

The levels of 2HG were measured in the three cell lines in the absence or presence of the mutant IDH1 inhibitor AGI-5198 (Xcess Biosciences Inc, USA) using tandem mass spectrometry. Briefly, 5–7 x 10^5^ cells were grown on a 100 mm petri dishes and left to attach. Cells were then treated with 0, 0.1, 0.5, 1 and 5 μM AGI-5198 as well as 5 μM DMSO in media with dialyzed fetal calf serum for 48 h. All samples were harvested at a non-confluent density. Metabolites were extracted from cell culture media and cell pellets using a modified version from Dang et al [16]. Briefly, 500 μl media from each sample was taken and mixed with 125 μl of HPLC grade methanol (Millipore, USA) at room temperature (80:20 medium:methanol). The mix was spun down at 3000 rpm for 20 min at 4°C and the supernatant was transferred to a cryo tube and kept in -80°C until analysis. In addition, cells were collected using 0.25% Trypsin (Corning, USA), washed once with phosphate buffered saline (PBS) (Lonza, USA) and counted for normalization. Metabolites were then extracted by immediate addition of 2 ml 80:20 methanol: water at -80°C and transferred to a dry-ice bed to simultaneously lyse cells and quench metabolism. The cell lysates were centrifuged at 14,000 g for 20 min at 4°C and the supernatant was transferred to a cryo tube and kept on -80°C until analysis.

D and L enantiomers were separated using a methanol gradient on a C18 column. 250 μL of 5 μM labeled D & L 2-hydroxyglutaric aciduria (Disodium (*RS*)-2-Hydroxy 1,5-pentanedioate-2,3,3-d_3_; cat # D-7496, CDN Isotopes, Canada) was added to 30 μL of cell lysate or media. A standard curve (0, 0.4, 2, 10, 50, 100 μM unlabeled D&L 2HG) was generated for quantitation (Sigma, USA). Samples were mixed and dried down at 50°C under a gentle stream of nitrogen. The samples were then derivatized at 75°C for 30 min with 50 g/L DATAN (diacetyl–L-tartaric anhydride; Sigma, USA) in 4:1 dichloromethane:acetic acid. Samples were again dried down at 50°C. Dried samples were reconstituted in HPLC water and run on an API4000 triple quadrupole (AbSciex, USA) with series 200 micropump HPLC pumps (Perkin Elmer, USA) in MRM mode using an Atlantis C18 3 μm x 2.1 mm x 150 mm column (Waters, USA) for chiral separation. Mobile phase buffer A was 90:10: acetonitrile:HPLC water with 10 mM tributylamine, pH 4.95. Samples were run using a 20–30% methanol gradient (Buffer B). Samples were run at a flow rate of 300 μL/min.

HPLC Gradient steps were:

Step 0 = 0 min 80%A, 20%B; Step 1 = 1 min 80%A, 20%B; Step 2 = 11 min 70%A, 30%B; Step 3 = 18 min 70%A, 30%B; Step 4 = 26 min 80%A, 20%B.

Instrument parameters:

Negative ion mode; Curtain gas pressure: 20 torr; Collision cell pressure: 4 torr; Ion spray voltage: -4000 V; Source temperature: 350°C; Gas 1: 45 psi; Gas 2: 40 psi; Declustering potential: -10 V; Entrance potential: -10 V; Collision energy: -7.5 V; Collision exit potential: -5 V; Labeled D&L 2HG Internal standard mass transition (m/z): 366.2 > 150.1; Unlabeled D&L 2HG mass transition (m/z): 363.2 > 147.1.

### Measurement of cell viability and colony-forming ability

Cell viability and colony-forming capacity were assessed by MTS assay (Cell-Titer 96 AQueous Non-Radioactive Cell Proliferation Assay, Promega Corporation, USA) and colony forming assay. Briefly, for MTS assay, 2000 cells per well were seeded on a 96-well plate and treated with different concentrations of AGI-5198 or DMSO (0, 5, 10, 20 μM) for 72 h. Cells were then incubated with MTS solution and absorbance was read at 490 nm using BioRad iMaxi plate reader (USA). Relative cell viability (%) was calculated as the mean absorbance of AGI-5198-treated cells relative to DMSO-treated cells. For colony forming assay, 100 cells per well were seeded on a 6-well plate in 2 ml media and treated with different concentration of AGI-5198 or DMSO (0, 1, 5, 10, 20 μM) for 6 days. Cells were washed, fixed in methanol, and stained with Toluidine Blue: Sodium tetraborate (Sigma, USA). Colonies were counted using ImageJ software.

### Measurement of cell migration

Scratch assay was used to assess the difference in migration distance between non-treated and AGI-5198-treated cells. Briefly, 1.5 x 10^5^ cells per well were seeded on a 6-well plate and grown until confluent. Two linear scratches were made in each well using a 1000 μl sterile pipette tip after removal of medium. Wells were washed with PBS to remove any cellular debris created by the scratch. Cells were then treated with specific concentration of AGI-5198 (0, 1, 5, 10, 20 μM) or 20 μM DMSO in 3 ml media. A time-lapse microscope system (Zeiss AxioObserver Z1 microscope, USA) was used to take pictures in three different fields in each sample every 20 min for 16 h. The migration distance was calculated as the distance between the scratch edges prior to treatment (time zero) and the time at which the scratch closed in the non-treated culture. The distances were measured using Axio Vision LA64 software.

### Apoptosis and cell cycle analysis

1 x 10^5^ cells per well were seeded on a 6-well plate in 3 ml media and treated with different concentrations of AGI-5198 (0, 1, 5, 10, 20 μM) or 20 μM DMSO for 72 h. Apoptosis was detected using Annexin V-FITC kit (Beckman Coulter, France) according to the manufacturer’s instructions. Cells stained with Annexin V-FITC and propidium iodide (PI) were analyzed by LSR-II flow cytometry (Becton Dickinson, USA). Apoptotic cells were quantified, including early apoptotic cells only positive for Annexin V and late apoptotic cells double positive for both Annexin V and PI. Data was analyzed by FlowJo-V10 software (FlowJo, USA).

For cell cycle analysis, non-treated or AGI-5198-treated cells were resuspended in 500 μl cold PBS. Cells were then fixed by addition of 4.5 ml 70% cold 200-proof ethanol (Sigma, USA) for at least 14 h at -20°C. The fixed cells were then spun down at 300 g for 10 min at 4°C and washed once using cold PBS. Cells were stained with propidium iodine with RNase (Cell Signaling Technology, USA) and analyzed using LSR-II flow cytometry. Data was analyzed by ModFit LTTM software (Verity Software House, USA).

### Biostatistics

Statistical analysis was performed by GraphPad Prism 6. Data was analyzed using one-tailed student’s unpaired t-Test. Data was denoted as Mean ± SEM (standard error of mean).

## Results

### DNA sequencing confirms IDH mutations in human chondrosarcoma cell lines and patient tumor

**Fig 2:**
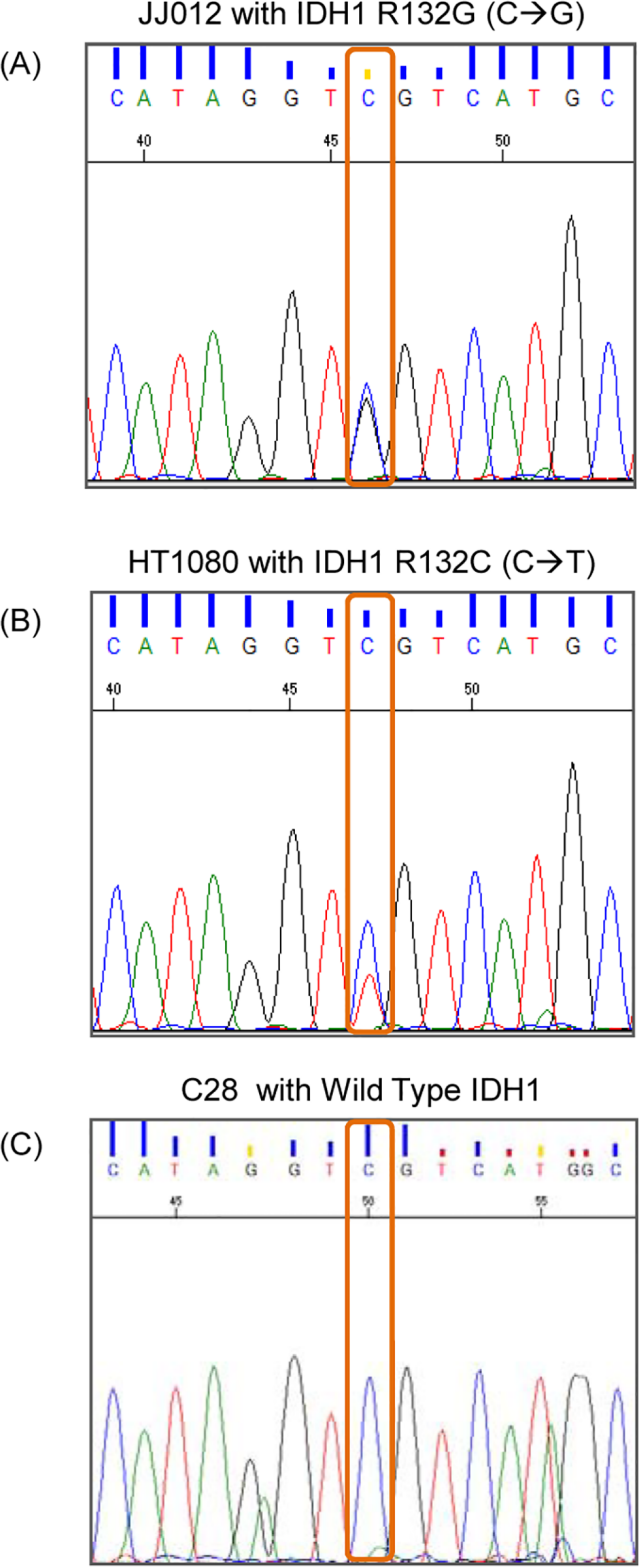
Human Chondrosarcoma Cell Lines JJ012 and HT1080 Harbor IDH1 Mutations. A: PCR-based sequencing confirmed that JJ012 encoded an R132G (CGT>GGT) missense mutation. B: HT1080 encoded an R132C (CGT>TGT) missense mutation. Both cell lines harbored an *IDH1* mutation in a single allele whereas the other allele was wild-type. C: The chondrocyte cell line C28 had no mutation in either allele.

**Fig 3:**
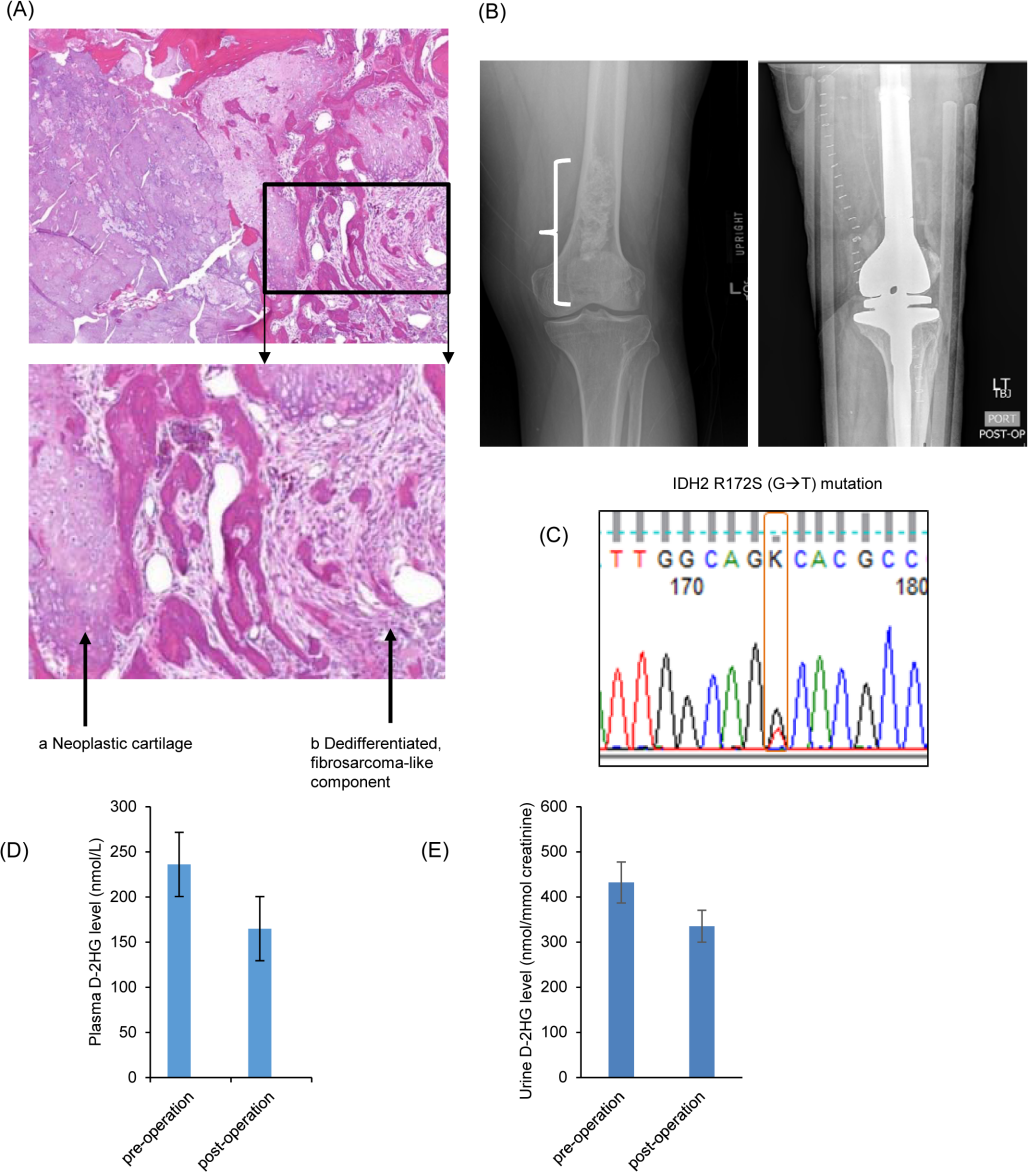
A Patient with IDH2 Mutant Dedifferentiated Chondrosarcoma Demonstrates a Drop in D-2HG Plasma and Urine Levels after Surgical Resection. A: Histologic image of this patient’s dedifferentiated chondrosarcoma showing neoplastic cartilage (Arrow a) juxtaposed to higher grade, and more cellular regions resembling fibrosarcomatous elements (dedifferentiated component, Arrow b.) B: plain radiograph pre- (left) and post-operation (right). Bracket delineates the tumor. C: PCR-based DNA sequencing showed that this patient’s tumor exhibits an IDH2 R172S (AGG>AGT) missense mutation. D: D-2HG levels measured in patient plasma samples showed a decrease after surgical resection. E: D-2HG levels measured in patient urine samples showed a decrease after surgical resection. Error bars depict one standard error of the mean (SEM) from three measurements.

In order to confirm whether the two human chondrosarcoma cell lines, JJ012 and HT1080, harbor the reported IDH mutations, we performed PCR-based DNA sequencing. We found that JJ012 DNA encoded an IDH1R132G (CGT>GGT) (Fig 2A) while HT1080 encoded an IDH1 R132C (CGT>TGT) mutation (Fig 2B). Both cell lines harbored an *IDH1* mutation in a single allele whereas the other allele was wild-type. The chondrocyte cell line C28 had no mutation in either allele (Fig 2C). For the patient with dedifferentiated chondrosarcoma of the distal femur who was undergoing resection (Fig 3A and 3B), sequencing performed by Knight Diagnostic Laboratories on the resected tumor revealed IDH2 R172S (AGG>AGT) mutation (Fig 3C).

### D-2HG levels decrease in human chondrosarcoma cells after treatment with a mutant IDH1 inhibitor, and after resection of the primary tumor

**Fig 4:**
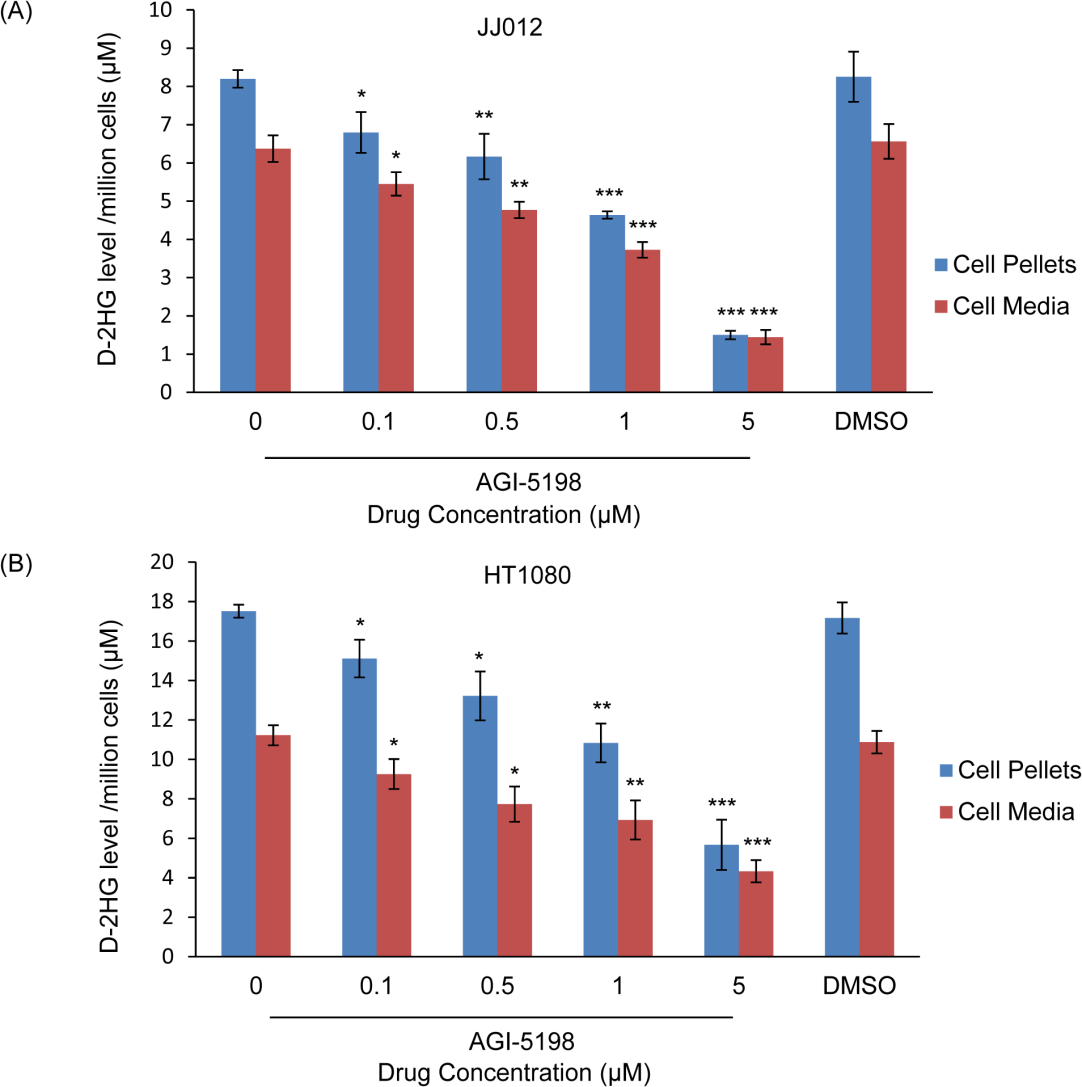
D-2HG Levels in AGI-5198 or DMSO-treated JJ012 and HT1080 Cell Pellets and Cell Culture Media. AGI-5198 reduced levels of D-2HG in both cell pellets and cell culture media of JJ012 (A) and HT1080 (B) in a dose-dependent fashion. Data was normalized by cell number and analyzed by one-tailed student’s unpaired t-Test between non-treated and drug-treated cells. Error bars depict SEM from 3 independent experiments. * *p*<0.05, ** *p*<0.01, *** *p*<0.001.

The oncometabolite D-2HG is detected in IDH-mutant cells including gliomas, and can be used as a biomarker for cancer cell activity in leukemias [6, 16, 17, 19, 24]. Thus, we hypothesized that D-2HG could be similarly measured in IDH1-mutant human chondrosarcoma cells, and would serve as a biomarker for tumor cell activity. First, we evaluated D-2HG levels by tandem mass spectrometry in IDH1-mutant chondrosarcoma cell lines and control wild-type IDH chondrocytes treated with AGI-5198 or DMSO. In JJ012 cell pellets, D-2HG levels decreased in a dose-dependent manner from 8.20 ± 0.23 μM in non-treated cells to 1.50 ± 0.11 μM after treatment with 5 μM AGI-5198 (*p*<0.001) (Fig 4A). Similarly, in HT1080 cell pellets, D-2HG levels decreased from 17.51 ± 0.33 in non-treated cells to 5.67 ± 1.28 after treatment with 5 μM AGI-5198 (*p*<0.001) (Fig 4B). D-2HG also decreased when measured in the media of both treated cell lines. Notably, the stereoisomer of D-2HG, L-2HG was undetectable in all samples (S1A Fig). The D-2HG level in IDH1-wild type chondrocyte cell line C28 was undetectable (S1B Fig). Thus, D-2HG appears to be measurable in chondrosarcoma cells and decreases as a biomarker for mutant IDH enzyme activity.

To explore this observation further, we next tested D-2HG levels in peripheral blood plasma and urine samples of a patient with dedifferentiated chondrosarcoma. As previously discussed, the patient’s tumor showed a mutation in IDH2 (Fig 3C). In plasma, D-2HG decreased from 236 ± 34.29 nmol/L prior to surgery to 165 ± 15.50 nmol/L one week after resection (Fig 3D). In urine, D-2HG decreased from 432 ± 45.50 nmol/mmol creatinine to 335 ± 35.31 nmol/mmol creatinine (Fig 3E). Although this is only one sample, and needs to be confirmed with multiple time points in additional patients, it supports the concept that D-2HG levels correlate with IDH-mutant chondrosarcoma cell activity.

### Treatment with mutant IDH1 inhibitor AGI-5198 inhibited colony formation in human chondrosarcoma cell lines

**Fig 5:**
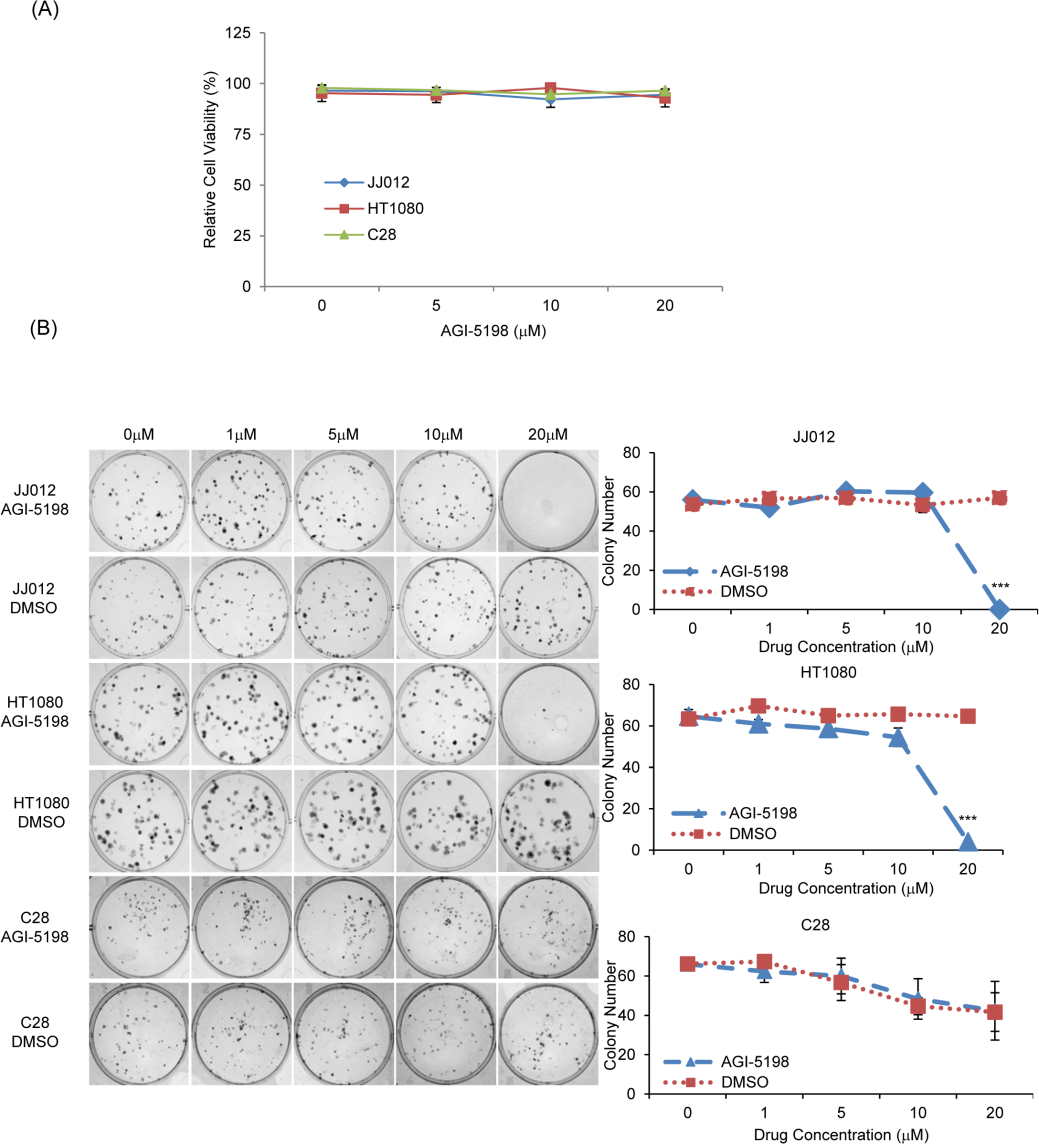
Treatment with AGI-5198 Impedes Colony Formation in IDH1-mutant JJ012 and HT1080 Human Chondrosarcoma Cells, While Sparing C28 Cells. A: Cell viability of JJ012, HT1080 and C28 cells. Cells were treated with increasing concentrations of AGI-5198 or DMSO and analyzed using MTS assay after 72 h of treatment. Relative cell viability (%) was calculated as the mean absorbance of AGI-5198-treated cells relative to DMSO-treated cells. B: Colony forming number of JJ012, HT1080 and C28 cells. Cells were treated with AGI-5198 or DMSO for 7 days, then the colonies were stained and counted. Error bars depict SEM from 3 independent experiments. *** *p*<0.001.

With evidence of AGI-5198 decreasing D-2HG production and suppressing the mutant IDH1 enzyme activity in chondrosarcoma cells, we next assessed whether this translated into inhibition of cell growth and tumorigenic activity. Effects of AGI-5198 on cell viability and colony forming capacity were assessed using MTS and colony forming assays. While the short-term MTS assay did not show any significant changes in the cell viability between AGI-5198-treated cells compared to non-treated alone (Fig 5A), AGI-5198 treatment at 20 μM completely inhibited colony formation in JJ012 cells (Non-treated 56 ± 0.58 vs. 0, *p*<0.001) and HT1080 (Non-treated 65 ± 3.28 vs. 0, *p*<0.001) (Fig 5B). Importantly, the same concentrations of AGI-5198 did not inhibit colony formation in IDH1-wild type C28 chondrocytes (Fig 5B). Thus, AGI-5198 appears to selectively inhibit longer-term tumor cell growth in IDH1-mutant chondrosarcoma cells while sparing non-malignant, IDH1 wild-type normal chondrocytes.

### High AGI-5198 treatment induces apoptosis and G2/M cell cycle arrest in human chondrosarcoma cell line JJ012

**Fig 6:**
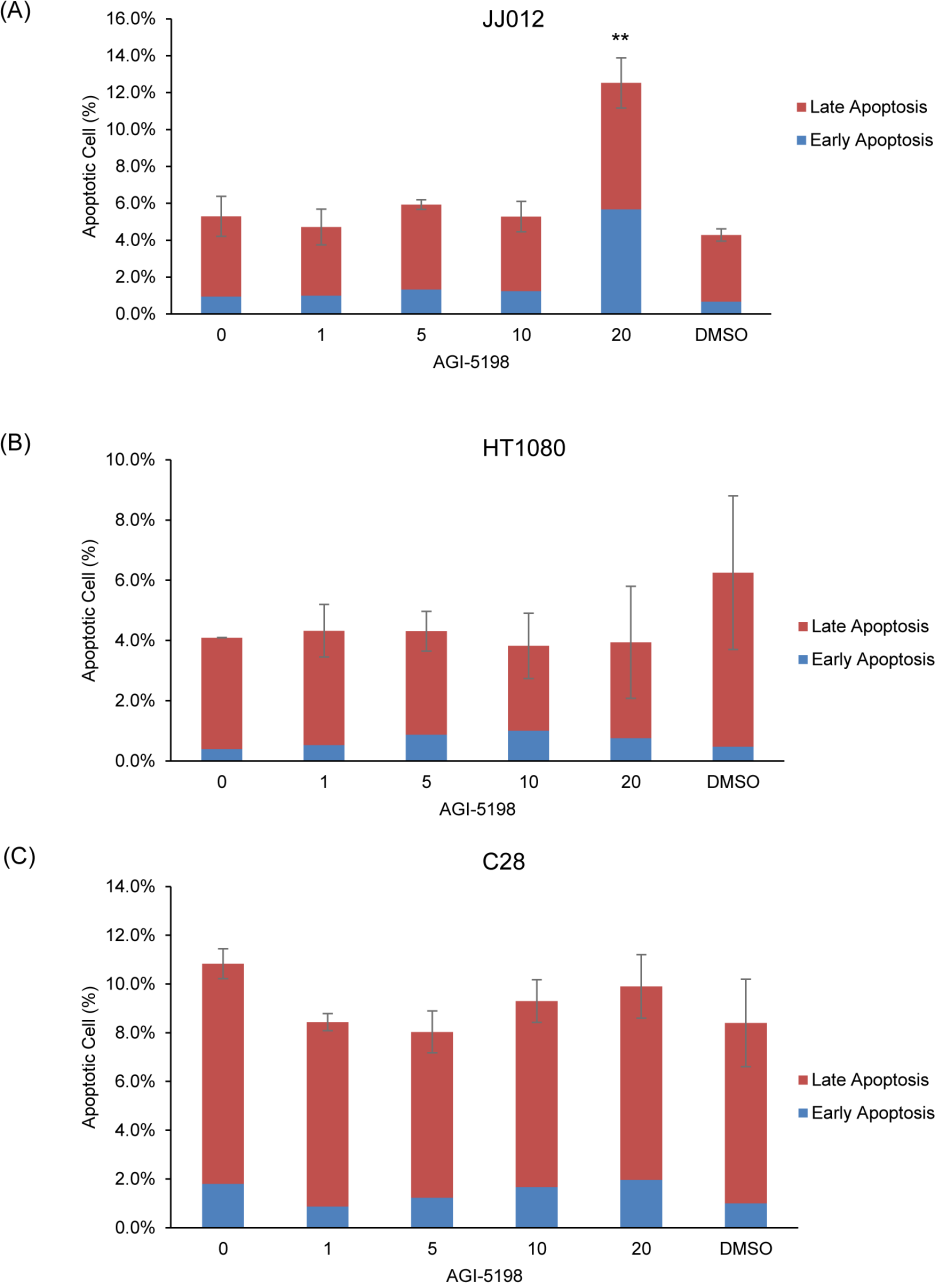
Apoptotic Cell Percentage in AGI-5198 or DMSO-treated JJ012 (A), HT1080 (B) and C28 (C) Cells. Data was analyzed by one-tailed student’s unpaired t-Test between non-treated and drug-treated cells. Error bars depict SEM of total apoptotic cells (early apoptotic cells + late apoptotic cells) from 3 independent experiments. Only *p* value of total apoptosis data analysis was shown here.* *p*<0.05, ** *p*<0.01.

Since AGI-5198 treatment inhibited colony formation, we next investigated whether treatment was inducing apoptosis, or leading to cell cycle arrest in a cytostatic fashion. Apoptosis was assessed in the three cell lines after 72 h of AGI-5198 treatment or DMSO alone by Annexin V staining followed by flow cytometry. A slight increase of total apoptotic cells (non-treated 5.2% vs 12.6%, *p*<0.01) including early apoptotic cells (non-treated 0.9% vs 5.7%, *p*<0.05) and late apoptotic cells (non-treated 4.3% vs 6.9%, *p*<0.05) was only observed at 20 μM AGI-5198-treated JJ012 cells (Fig 6A). However, no increase in apoptosis was observed in AGI-5198-treated HT1080 (Fig 6B) or C28 (Fig 6C) cells.

**Fig 7:**
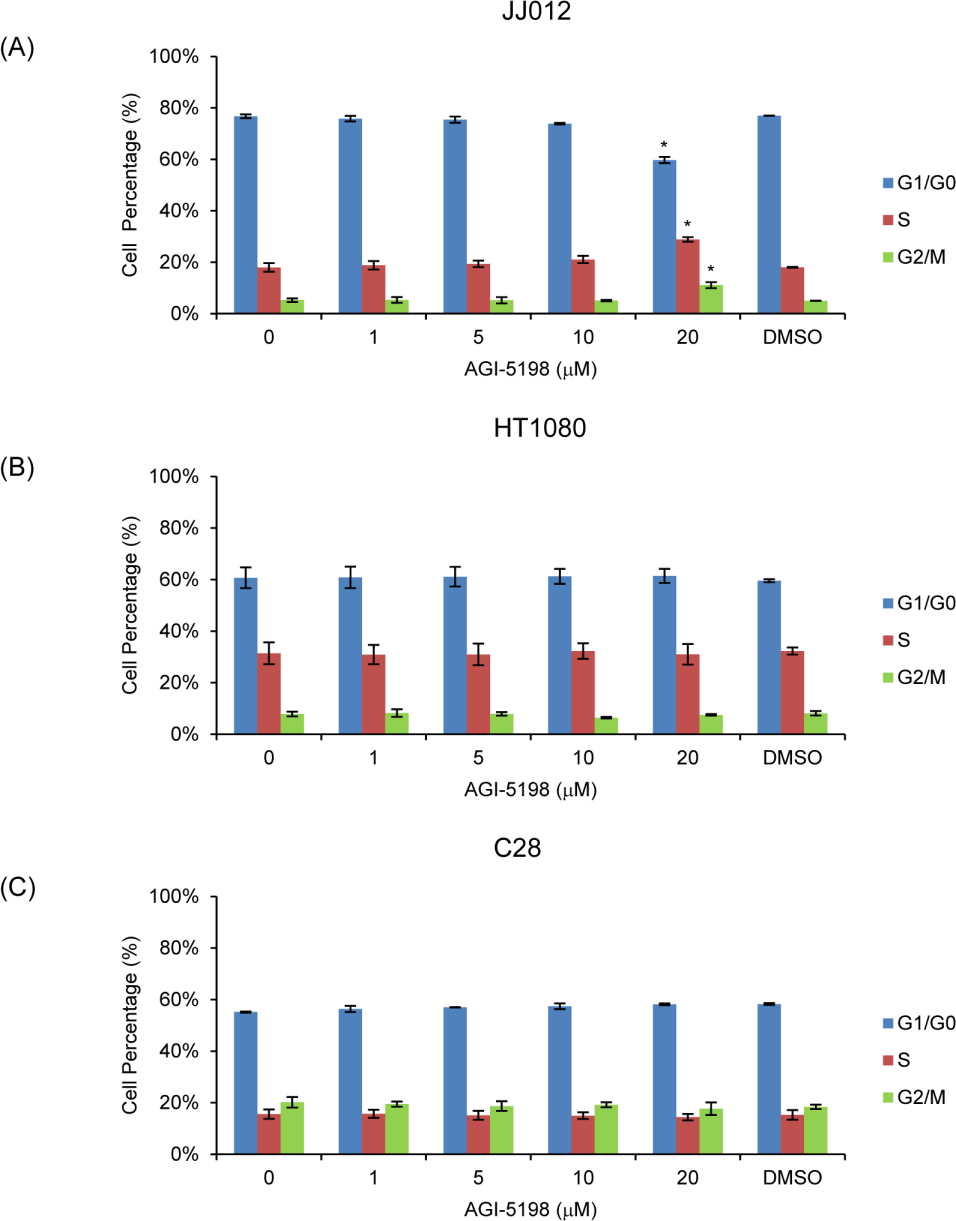
Cell Cycle Changes in AGI-5198 or DMSO-treated JJ012 (A), HT1080 (B) and C28 (C) Cells. Data was analyzed by one-tailed student’s unpaired t-Test between non-treated and drug-treated cells. Error bars depict SEM from 3 independent experiments. * *p*<0.05.

Next we assessed the effects of mutant IDH1 inhibition by AGI-5198 on the cell cycle. After treatment with AGI-5198 or DMSO for 72 h, JJ012, HT1080 and C28 were stained for propidium iodide and analyzed by flow cytometry to determine cell cycle phases. In JJ012 cells treated with 20μM AGI-5198, we observed 17% decrease in G1/G0 phase (*p*<0.05), 11% increase in S phase (*p*<0.05) and 6% increase in G2/M phase (*p*<0.05) compared to non-treated cells (Fig 7A). These results combined with the similar proliferative cell number during cell culture in non-treated and AGI-5198-treated cells suggests that AGI-5198 induced a G2/M cell cycle arrest. However, we did not see any changes in cell cycle distribution in AGI-5198-treated HT1080 (Fig 7B) or C28 (Fig 7C) cells.

### AGI-5198 significantly inhibited migration distance in human chondrosarcoma cell lines

**Fig 8:**
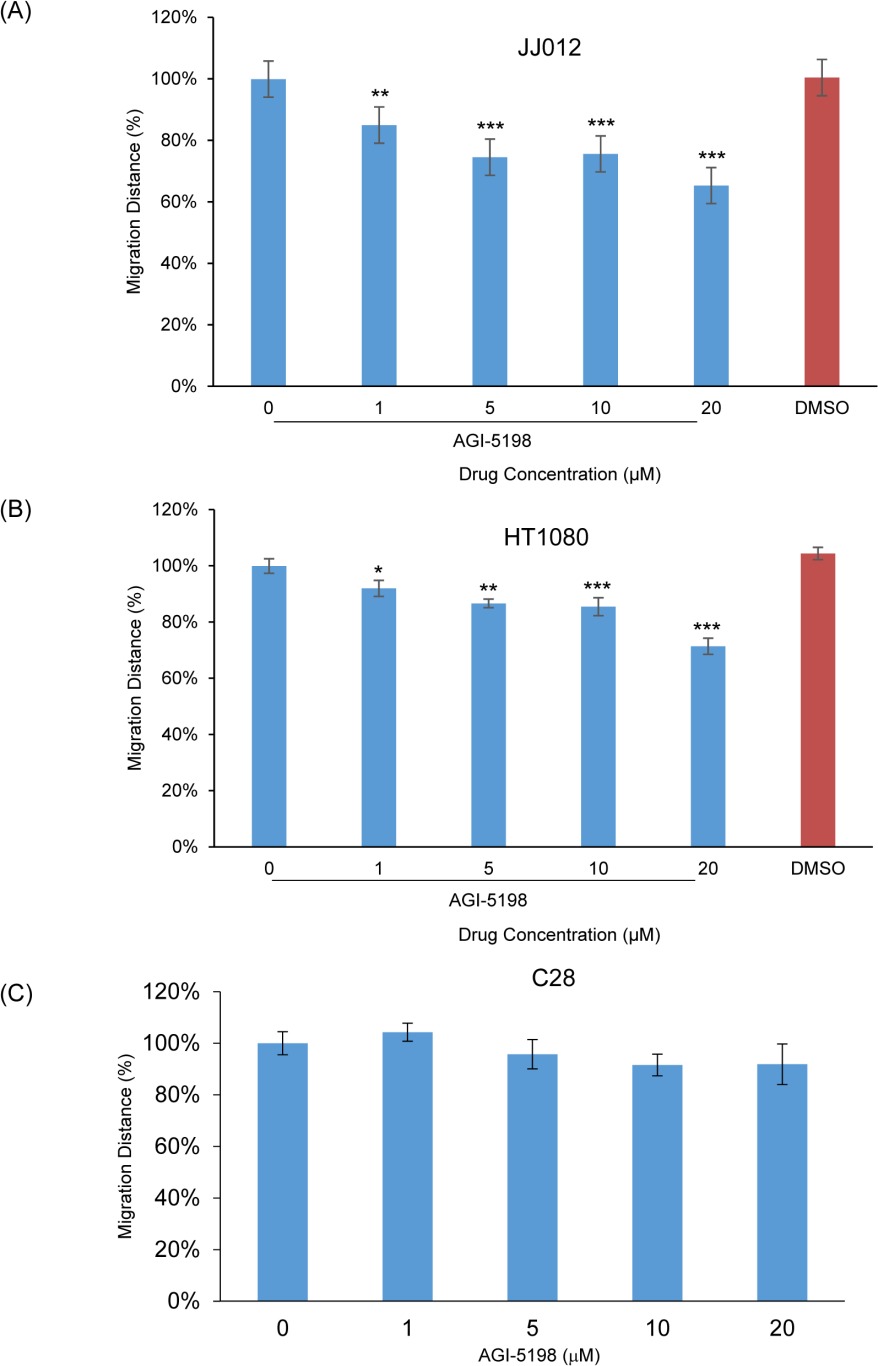
Migration Distance in AGI-5198 or DMSO-treated JJ012 (A), HT1080 (B) and C28 (C) Cells. The migration distance was calculated as the distance between the scratch edges prior to treatment (time zero) and the time at which the scratch closed in the untreated cells. Data was normalized as the percentage of non-treated cells in migration distance. Data was analyzed by one-tailed student’s unpaired t-Test between non-treated and drug-treated cells. Error bars depict SEM from 3 independent experiments. * *p*<0.05, ** *p*<0.01, *** *p*<0.001.

Although IDH1 inhibition completely inhibited colony forming activity in chondrosarcoma cells, this did not seem to be completely explained by the modest increase in apoptosis nor by cell cycle arrest. Thus, we investigated whether IDH1 blockade was inhibiting other tumorigenic functions. First, we assessed cell migration by scratch assay in the three cell lines after treatment with AGI-5198. We found a dose-dependent inhibition of migration with AGI-5198 treatment compared to non-treated cells in both chondrosarcoma cell lines. Migration distance decreased by up to 35% (*p*<0.001) relative to non-treated cells in 20 μM AGI-5198-treated JJ012 cells (Fig 8A), and up to 29% (*p*<0.001) relative to non-treated cells in 20 μM AGI-5198-treated HT1080 cells (Fig 8B). In contrast, migration distance did not decrease in AGI-5198-treated C28 cells relative to non-treated cells (Fig 8C).

## Discussion

Chondrosarcoma is the second most common primary malignant tumor of the skeleton, and is particularly problematic since most subtypes are resistant to chemotherapy or radiotherapy. Thus, development of novel treatments are critical to patients with tumors that are not amenable to surgical resection. The recent discovery that many chondrosarcomas have a mutation in IDH enzymes offers hope that targeted IDH inhibitors may provide a novel treatment strategy and improve outcomes for patients.

The work of Rohle et al and Fathi et al first demonstrated the relationship between these mutations and tumorigenesis, and established D-2HG as a biomarker for IDH-mutant cancer cell activity [24, 38]. Using AGI-5198, Rohle and colleagues demonstrated that mutant IDH1 inhibition blocked production of D-2HG by the mutant enzyme in a dose-dependent manner, and inhibited tumor growth in mouse xenografts and promoted cell differentiation in glioma cells [38]. In our study, we aimed to demonstrate that AGI-5198 would have similar effects on IDH1-mutant human chondrosarcoma cell lines JJ012 and HT1080. We found that AGI-5198 decreased the levels of D-2HG within cells and the conditioned cell medium in a dose-dependent manner in the two chondrosarcoma cell lines. In fact, D-2HG level was undetectable after treatment with 10μM or 20μM AGI-5198. This is consistent with Rohle’s findings which documented that AGI-5198 decreased D-2HG level dose-dependently in R132H-IDH1 mutant TS603 glioma cells. These results provide direct evidence that this compound, identified to inhibit mutant R132H IDH1 enzyme, is also able to inhibit R132G and R132C mutations in IDH1. L-2HG, the stereoisomer of D-2HG, was not detectable in any of our samples, consistent with previous observations that L-2HG is unlikely contributing to tumorigenesis from mutant IDH1. Thus, this supports our hypothesis that D-2HG can serve as a biomarker of IDH-mutant enzyme activity in chondrosarcomas.

In further support of this concept, we observed a decrease in D-2HG levels in post-resection blood and urine samples of a patient with IDH2 R172S mutated dedifferentiated chondrosarcoma. Interestingly, the level did not fall to undetectable which has been demonstrated in IDH-mutant leukemia patients achieving complete remission after chemotherapy [24]. Further investigations will be needed to determine whether this could be a result of prolonged clearance from the patient, limitations in the assay, or whether the patient could still have occult micrometastatic disease. The upcoming clinical trials of mutant IDH1 inhibitor AG-120 in solid tumors which include measurement of D-2HG levels will likely shed light on these important clinical questions.

While this decrease in D-2HG supported that the mutant IDH1 enzyme was being inhibited with AGI-5198 treatment in our chondrosarcoma cell lines, the next question was if we could demonstrate corresponding anti-tumor activity with the compound, similarly to the results seen in gliomas. Interestingly, we did not see short-term inhibition of cell viability or profound early induction of apoptosis with AGI-5198 treatment in chondrosarcoma cells. However, significant inhibition in long-term tumorigenic activity was witnessed based on colony-forming potential. Additionally, the migration of these cells was significantly altered, suggesting that inhibition of mutant IDH1 is clearly disrupting chondrosarcoma cell physiology.

Regarding possible mechanisms to explain our observations, previous studies have reported altered cell differentiation via effects on methylation status after mutant IDH inhibition in cancer cell lines. For example, Wang et al showed that targeted inhibition of mutant IDH2 in leukemia cells reversed the histone and genomic DNA methylation state and induced cellular differentiation [42]. Rohle et al found under conditions of near-complete D-2HG inhibition by AGI-5198, the mutant IDH1 inhibitor induced demethylation of histone H3K9me3 and expression of genes associated with gliogenic differentiation [38]. Based on these findings, we hypothesize that AGI-5198 preferentially affects migration due to the induction of wide scale epigenetic changes, possibly leading to cell differentiation. Given the relatively slow cell cycle of chondrosarcoma cells, and the well-reported phenomenon of the need for numerous cell cycles for epigenetic alterations to show effects, this would explain why we were not seeing profound changes in chondrosarcoma cell growth in the relatively short term assays used in this exploration. The investigation into AGI-5198-mediated changes in gene expression, epigenetics and differentiation of IDH-1 mutant chondrosarcoma cell lines is ongoing in our laboratory.

Most cancer chemotherapeutics have historically relied on induction of apoptosis or interruption of the cell cycle, generally through production of DNA damage that then triggers cell cycle arrest and apoptotic signaling [2]. With the new development of targeted therapeutics like mutant IDH, anti-tumor effects are exerted through alternative pathways, such as disruption of cancer cell metabolism. Thus, this would explain why our results showed only minimal impact on the induction of apoptosis and only slight cell cycle changes with AGI-5198 treatment. A lack of early decrease in cell viability after 72 h of incubation was also reported in early preclinical testing of AG-120 in human leukemia cells (Agios investigator’s brochure). Additionally, despite not producing cell death at lower concentrations, our results confirmed that AGI-5198 significantly decreased the migration distance at concentration much lower concentration of 1 μM in JJ012 and HT1080. This could suggest the drug is particularly potent at inhibition of the metastatic phenotype of chondrosarcoma cells. Our interpretation of these and other’s results is that investigation of pre-clinical effects of mutant IDH inhibitors will likely require alternative assays focusing on longer-term outcomes rather than the traditional rapid response seen in more potent chemotherapeutics that induce apoptosis. Increased tumor latency in xenograft models, and ultimately the response in human clinical trials showing prolonged time to progression may be the more accurate way to assess the therapeutic potential of mutant IDH inhibition and similarly directed novel therapies.

In conclusion, our study investigated *in vitro* characteristics of IDH-mutated human chondrosarcoma cells and demonstrated anti-tumor activity of a mutant IDH1 inhibitor, AGI-5198. As mentioned before, mutant IDH enzymes fail to convert isocitrate into -KG and instead gain the function to catalyze α-KG into the oncometabolite D-2HG. 2HG, at high levels, inhibits α-KG-dependent dioxygenases including epigenetic regulators, which results in altered epigenetic state of DNA and histone, and promotes tumorigenesis [16, 26, 27, 43]. Therefore, we believe that is why the anti-tumorigenic activity of AGI-5198 was most obvious at high drug concentrations at which the 2HG production was nearly completely blocked. Our data supports that D-2HG is likely to serve as a biomarker for mutant IDH activity in chondrosarcomas, and supports additional investigation of mutant IDH1 inhibition as a treatment strategy in patients with unresectable disease. Future work will be directed at unraveling the complex mechanisms of mutant IDH activity in chondrosarcoma and may improve understanding of how to best utilize therapies that impair cancer cell metabolism, rather than induce rapid apoptosis. Clinical trials investigating mutant IDH inhibitors in solid tumors with IDH mutations have the potential to drastically change standard therapy for chondrosarcomas, and unlock an entirely novel approach to treatment of cancers.

## Supporting Information

S1 Fig Representative D&L-2HG Chromatograms from Non-treated JJ012 (A) and C28 (B) Cells. Neither D-2HG or L-2HG is detectable in C28 cells. In contrast non-treated JJ012 cells show high levels of D-2HG (left peak), but not L-2HG (right peak). Similar results could be seen in HT1080 cells. Labeled D&L 2HG internal standard peaks are shown in red and unlabeled amounts of D&L 2HG from the samples are shown in blue (Arrow).
(TIF)
