# Supplementary figures and images for: Treatment with a Small Molecule Mutant IDH1 Inhibitor Suppresses Tumorigenic Activity and Decreases Production of the Oncometabolite 2-Hydroxyglutarate in Human Chondrosarcoma Cells
Source: PLoS One. 2015 Sep 14;10(9):e0133813. doi: 10.1371/journal.pone.0133813 (PMC4569544; doi:10.1371/journal.pone.0133813)

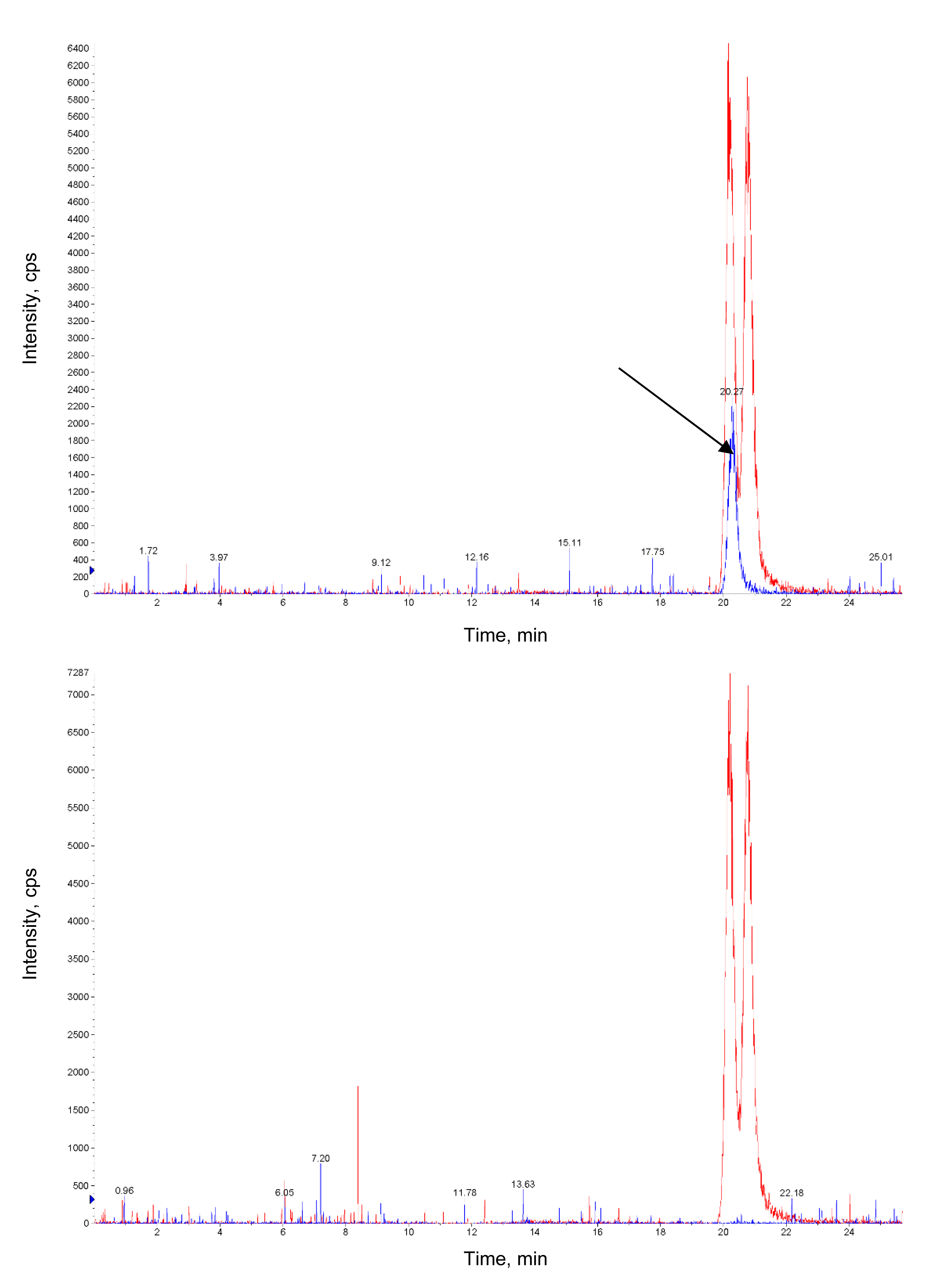

Supplement: S1 Fig — Neither D-2HG or L-2HG is detectable in C28 cells. In contrast non-treated JJ012 cells show high levels of D-2HG (left peak), but not L-2HG (right peak). Similar results could be seen in HT1080 cells. Labeled D&L 2HG internal standard peaks are shown in red and unlabeled amounts of D&L 2HG from the samples are shown in blue (Arrow). (TIF) [file pone.0133813.s001.tif]
